# Spring brings together the European Academies of Medicine in Bucharest for the 2010 F.E.A.M. Conference

**Published:** 2010-05-25

**Authors:** VL Purcarea

**Affiliations:** ‘Carol Davila’ University of Medicine and PharmacyRomania

The Federation of European Academies of Medicine (F.E.A.M.), founded in 1993 in Brussels, has the goal of promoting cooperation between national Academies of Medicine. It is the main association functioning as a bridge between the political and administrative authorities of the European Union and the National Medical Academies of Europe, in their role as advisors with concern to medicine and public health. Since the promulgation of its status in the Royal Decree on 31 March, 1995, F.E.A.M. has been given the full rights of an international association with a scientific objective. Its head office is still located in Brussels, in the prestigious Palais des Academies. F.E.A.M. has grouped leading European medical experts for its work, having three main objectives, as stated in their statute: 

**Figure 1:**
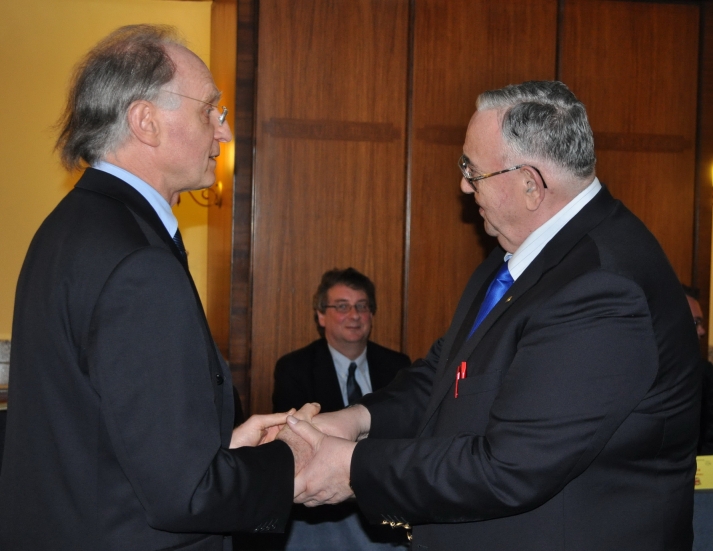
Prof.  H.E. Blum (L) and Acad. Prof. L.M. Popescu (L)–The current and future Presidents of the Federation of European Medical Academies are responsible with setting the direction of future medical research.

- To identify public health issues common to the Member States and to debate them in its conferences organized for the scientific Delegates of its Member Academies;
- To produce short reports based on an objective and impartial analysis of these issues which are submitted to the European Union and the Member Academies;
- To facilitate contacts between Member Academies and other organizations, with respect to the activities of the European Union, in matters concerning medicine and public health.

**Figure 2:**
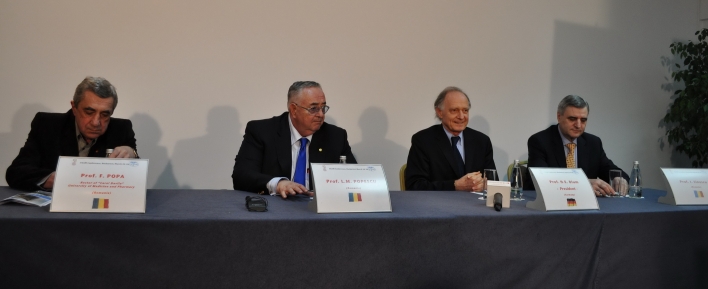
Prof. Florian Popa, Member of the Romanian Parliament and Rector of ‘Carol Davila’ University of Medicine and Pharmacy in Bucharest, Acad. Prof. L.M. Popescu, Prof. H.E. Blum and Prof. Ioanel Sinescu held the press conference after the FEAM administrative session

This year, F.E.A.M. has given the Romanian Academy of Medical Sciences the honor to host and organize the Spring Conference, during the 24^th^ and the 26^th^ of March, in Bucharest. Past conferences were held in Prague, Paris, Brussels, Rome and Lisbon. For the first time in the whole existence of FEAM, representatives from out of Europe, from countries such as China, USA, and Israel have been invited to participate. 

This conference had two main objectives, both fulfilled with great success. The first objective, an administrative one, was to elect a new President–to–be for F.E.A.M., who was found in the person of Academician Prof. Dr. Laurentiu Mircea Popescu, President of the Romanian Academy of Medical Sciences. After his election, he will enjoy the Vice–President, President and Past President positions, each for two years. The second objective, an academical one, was to outline the high–end research and opinions in two mainstream domains of medicine: Translational Cardiology, on one part, and Medical Bioethics and Public Health on the other.

**Figure 3:**
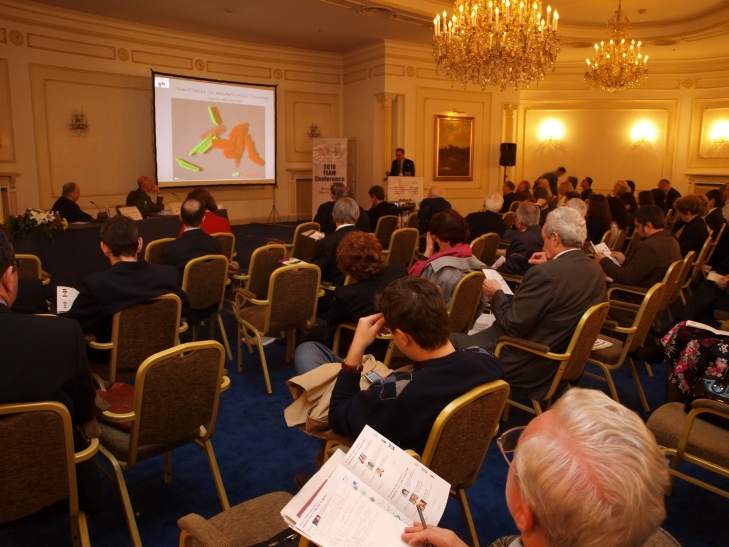
The main conferences were held in Bucharest, in front of a very small, but selected auditory, and in a highly interactive and open–minded manner.

Selected speakers from twelve countries and three continents have presented their point of view during the two major scientific sections of the conference. The first section entitled ‘Translational Cardiology: from Molecular and Cellular Cardiology to Bedside’, brought light on the latest scientific breakthroughs of this field. The program included subjects such as *‘The embryonic epicardium: an essential element of cardiac development’* by Prof. R.M. Chapuli from Spain, *‘The role of the epicardium derived cells in cardiac development and repair’* by Prof. Adriana C. Gittenberger–de Groot from Netherlands, *‘From genes to function: Single nucleotide polymorphisms as markers for cardiac disease’* by Prof. C. deWit from Germany, *‘The role of endogenous carbon monoxide and heme oxygenase system in myocardium’* by Prof. A. Tosaki from Hungary, *‘Regenerative Medicine: a cardiac surgeon's view’* by Prof. S.S.Hu from China *‘Relationships between telocytes and cardiomyocytes during pre– and post–natal life’* by Prof. Maria–Simonetta Faussone–Pellegrini and Prof. D. Bani from Italy, *‘Myocardial telocytes: a new distinct cellular entity’* by Prof. S. Kostin from Germany, *‘Cardiac stem cells, from science to therapy’* by Prof. P. Doevendans from Netherlands, *‘Cardiospheres and tissue engineering for myocardial regeneration: potential for clinical application’* by Prof. G. Frati from Italy, *‘Redox signaling of cardiac stem cells’* by Prof. D. K. Das from S.U.A., *‘Vascular Disease: A Critical Stage in the Vascular Continuum’* by Prof. Maya Simionescu from Romania, *‘Use of Cell and Gene Therapy for Engineering Blood Vessels’*  by Prof. M. Flugelman from Israel and *‘Developmental origins of hypertrophic cardiomiopathy phenotypes’* by Prof. F. Cecchi from Italy.

**Figure 4:**
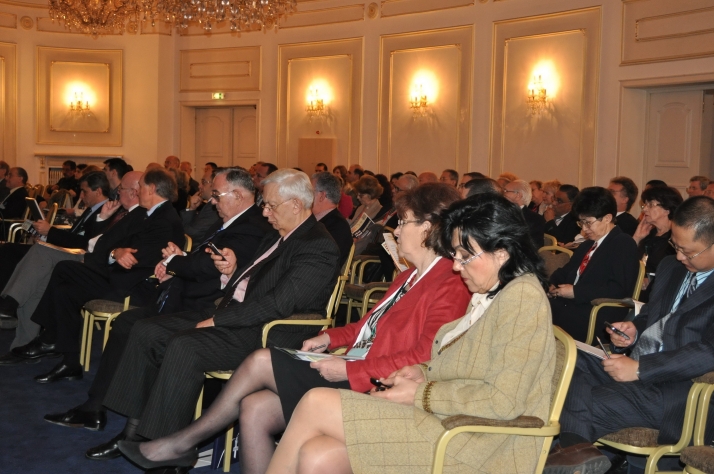
The conference brought together the best doctors in the fields of Cardiology and Bioethics and Public Health. The international exchange of experience and ideas was the mainstream of the event

A high importance was given to the newly discovered Interstitial ‘Cajal–like’ Cells (ICLCs) telocytes and their importance in Cardiology. Both Prof. G. Frati from Italy and Prof. S. Kostin from Germany focused on the importance of telocytes in Regenerative Cardiology, receiving valuable feedback from Prof. L.M. Popescu. The interactivity of the conference was of great significance, since it directed research towards new grounds, and stands as the fundamental source of new ideas and new perspective in a field that is responsible for the caring of more and more patients worldwide.

The second section of the conference focused on Medical Bioethics and Public health, with presentations such as *‘The medical profession in transition: what makes a good doctor?’* by Prof. H.E. Blum from Germany, *‘Reconciling Bioethics with Public Health’* by Sir Peter Lachmann, Prof. from the United Kingdom, *‘Skylla and Kharybdis: stem cell researchers squeezed between advancement of science and self promotion?’* by Prof. Y. T. Konttinen from Finland, *‘Ethical issues in Tissue Engineering and Molecular Medicine’* by Prof. R.E. Horch also from Germany, *‘Bioethics, a basic discipline in public health’*  by Prof. A. Knottnerus from The Netherlands, and *‘Bioethics of Patient Cell Reprogramming: In Search for Post JAK2 V617F Events in Blood Cancers’* by Prof. St.N. Constantinescu from Belgium.

The final presentation and closing remarks were summarized in an impressive presentation regarding *‘The New Medicine–a general cultural project and a perspective in civilization’* by Prof. C.T. Dragomir from Romania. 

High importance was given to the conference by the Romanian administrative, legislative and academic world, with attendance of Daniel Petru Funeriu the Minister of Education and Culture, Adrian Streinu–Cercel, Secretary of State in the Ministry of Health, Florian Popa, Member of the Romanian Parliament and Rector of the University of Medicine and Pharmacy in Bucharest, Mircea Cinteza, Senator, Stefan Iosif Dragulescu, Deputy and Rector of the University of Medicine in Timisoara and Academician Ion Ababii, Rector of the University of Medicine in Chisinau, as well as other personalities transforming this event into a base–stone for future academical, social and cultural perspectives in Romanian health and education.

To quote Acad. Prof. L.M. Popescu in his presentation of the event, we sincerely believe that Bucharest has been the heart of translational cardiology and medical bioethics, even for just a couple of days.

**Figure 5:**
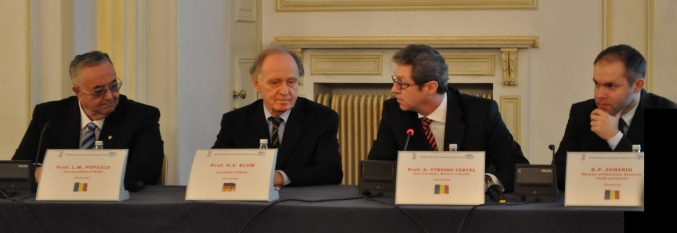
Prof. L.M. Popescu, Prof. H.E. Blum, Secretary of State A. Streinu–Cercel and Ministry of Education and Culture  D.P. Funeriu – an exchange of opinions and ideas in setting the future course of the Romanian Medical and Research fields.

**Figure 6:**
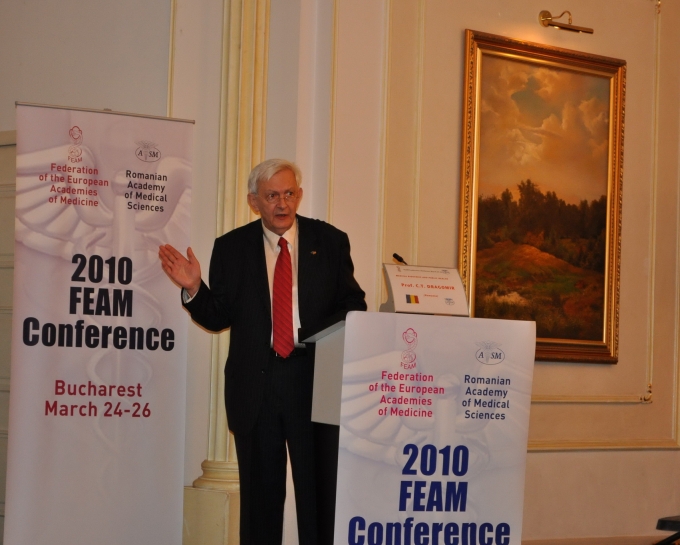
Prof. C.T. Dragomir presenting the final considerations and summarizing the event in his presentation: ‘The New Medicine – a general cultural project and a perspective in civilization’

